# Laboratory Performance of Hot Mix Asphalt with High Reclaimed Asphalt Pavement (RAP) and Fine Reclaimed Asphalt Pavement (FRAP) Content

**DOI:** 10.3390/ma12162536

**Published:** 2019-08-09

**Authors:** Sen Han, Xianpeng Cheng, Yamin Liu, Yacai Zhang

**Affiliations:** 1 Highway and Airport Pavement Research Center, School of highway, Chang’an University, Xi’an 710064, China; 2 Kunming Engineering Corporation Limited under Power China, Kunming 650051, China

**Keywords:** recycled mixtures, RAP, fine reclaimed asphalt pavement, low-temperature performance, fatigue performance, moisture performance

## Abstract

Recently, there has been an increasing interest in the applications of recycled mixtures with a high reclaimed asphalt pavement (RAP) content. However, many government departments are hesitant about the applications of that due to being worried about the durability of its pavement, and few findings has been reported in terms of the percentage of fine reclaimed asphalt pavement (FRAP, 0~5 mm) in recycled mixtures. In this paper, 25% or more RAP by the weight of aggregates is defined as high RAP and high FRAP refers to 10% or more FRAP by the weight of aggregates. This paper examines the laboratory performances of mixtures with high RAP (30%, 40%, and 50%) and FRAP (10%, 15%, and 20%). Performance evaluations have been conducted by conventional tests, including the low-temperature bending test, fatigue test, and the moisture susceptibility test. The results show that with the increasing RAP and FARP contents, 41% (30-R-10) to 63% (50-R-15) of virgin asphalt can be saved, both the low-temperature and fatigue performance decrease, and the moisture performance firstly increases and then decreases. The results of analysis of variance (ANOVA) in terms of low-temperature and moisture performance show that RAP contents in recycled mixtures have a significant effect on the performance, while the effect of FRAP contents on the performance of recycled mixtures is insignificant.

## 1. Introduction

Asphalt pavement is widely used in the world because of its good benefits, such as its safety, low noise, easy maintenance, easy recycling, and etc. More than 260 million tons of hot and warm mixed asphalt mixtures were produced in Europe every year from 2008 to 2017 [1]. There were a large quantity of removed pavement materials containing aged asphalt and aggregates produced per year because the service of asphalt pavement is usually 10–15 years. Reprocessing of these materials is referred to as reclaimed asphalt pavement (RAP).

Over the past three decades, RAP has been extensively studied due to its economic and environmental benefits [2,3,4,5,6]. Aggregates and asphalt binder are nonrenewable resources. Their amounts can be reduced during the production of hot mix asphalt (HMA) by applying RAP to a new pavement. Additionally, the use of RAP will reduce construction debris needed to be dealt with. Ultimately, using RAP makes the best utilization of natural resources and promotes the sustainable development of the asphalt industry.

Recently, there has been an increasing interest in the applications of recycled mixtures with a high RAP content [7]. It should be noted that 25% or more RAP by the weight of HMA is defined as high RAP. There are 35 state agencies in the USA allowing up to 29% RAP to be applied in the intermediate layer. Similarly, 20 state agencies use up to 29% RAP in the surface layer. However, few state agencies are actually doing it. Only 10 state agencies actually use up to 29% RAP in the intermediate layer, and the number of state agencies who use up to 29% RAP in the surface layer is less than 5 [8].

At high RAP contents, the amount of blending which occurs between RAP binder and the used virgin binder must be taken into account. Three different blending cases with two RAP contents (10% and 40%), black rock (no blending), actual practice, and total blending (100%) were conducted to evaluate the properties of the recycled mixtures [2]. The results showed that the differences among these three cases at 10% RAP could be ignored. At high RAP (40%), the differences between the black rock case and the other two cases became significant, and there was no significant difference between the actual practice and the total blending cases. This indicated that blending between the RAP binder and new binder actually occurred. Subsequently, other studies have demonstrated that partial blending rather than total blending occurred [3].

Laboratory performances of HMA with high RAP contents have been investigated. Higher RAP contents resulted in a higher susceptibility for low-temperature cracking of its recycled mixtures [9]. With increasing RAP contents, the fatigue performance of a fine aggregate matrix decreased, but the modulus increased. Additionally, rejuvenator helped improve the fatigue performance of a fine aggregate matrix [10]. The difference of the complex modulus between HMA containing 20% RAP and HMA without RAP was not significant. When HMA contained 40% RAP, the modulus of its recycled mixture was 49% higher than that of the virgin mixture [11]. The findings from laboratory performances have indicated that with higher RAP contents, both fatigue and low-temperature performances became worse.

Conversely, the long-term performance of pavements with high RAP has been very positive. Eighteen test projects built between 1989 and 1998 have been investigated [12]. Seven parameters from these pavements were examined, involving the International Roughness Index (IRI), rutting, fatigue cracking, longitudinal cracking, transverse cracking, block cracking, and raveling. The findings showed that overlays containing 30% RAP performed similarly to overlays involving a virgin mixture in terms of IRI, rutting, block cracking, and raveling. Field performances of pavements containing up to 50% RAP with different climates and traffic were examined. Overlays involving 30% RAP were found to perform as well as overlays with virgin mixtures [13].

The gradation of the RAP is significantly different from RAP aggregates because RAP is composed of aged asphalt binder and aggregate. For high RAP contents, it is necessary to divide RAP into two or more stockpiles. Fractionation of RAP helps control the quality of the recycled mixtures [14]. The RAP is usually divided into two stockpiles, coarse and fine RAP, representing the extremes of fractionation. Coarse RAP refers to fractionation RAP with a size lager than 4.75 mm, and the fractionation RAP passing through the 4.75 mm sieve is fine RAP (referred to hereafter as FRAP). The asphalt contents of FRAP are higher than those of coarse RAP, and FRAP is more easily heated [15]. An economic analysis of mixtures with different percentages (50% and 100%) of coarse RAP, FRAP, and total RAP was investigated. For the same percentage, the mixtures with total RAP had a lower construction cost than that with fine RAP, and the mixtures with coarse RAP had the highest construction cost [16]. Additionally, fractionation of RAP helps to increase the RAP content in recycled mixtures. Texas uses up to 10%, 20%, and 30% unfractionation RAP in surface, intermediate, and base layers, respectively. However, for fractionation RAP, the allowed RAP percentages used in these layers increases to 20%, 30%, and 40%, respectively [8].

The production cost of asphalt mixtures includes the materials, processing, trucking, and lay down cost, of which the materials cost is the most expensive, accounting for about 70% of the total cost. With a limited materials and finances supply, it is necessary to increase the RAP content. Moreover, few findings have been reported in terms of the high percentage of FRAP in recycled mixtures. FRAP is the most worth recycling in that it contains the highest asphalt content among RAP.

In this paper, 25% or more RAP by the weight of aggregates is defined as high RAP and high FRAP refers to 10% or more FRAP by the weight of aggregates. In order to explore the possibility of recycled mixtures incorporating high RAP and FRAP contents, this paper examined laboratory performances of mixtures with three RAP percentages (30%, 40%, and 50% by the weight of total aggregates), and there were also three FRAP rates (10%, 15%, and 20% by the weight of total aggregates) for each RAP content. Performance evaluations were conducted by conventional tests, including the low-temperature bending test, fatigue test, and moisture susceptibility test.

## 2. Materials and Methods

### 2.1. Materials

#### 2.1.1. RAP

RAP selected in this study came from the Bai-lan section of the G6 Beijing-Tibet Expressway. The separation of RAP was done on site. A mechanical sieving device with three sieve sizes (19 mm, 9.5 mm, and 4.75 mm (No. 4)) was used for the separation of RAP in an inclined position. Subsequently, the three RAP fractions 0–5 mm (FRAP), 5–10 mm, and 10–20 mm could be gained. The gradations of RAP are shown in Table 1.

RAP binder was obtained by solvent extraction and tested according to Chinese specification JTG F41-2008 [17]. Asphalt contents were determined according to AASHTO T319 [18]. Asphalt contents and physical properties of the aged asphalt binder among RAP are shown in Table 2.

From Table 2, it can be seen that the asphalt content of FRAP is 1.6 times that of RAP (5–10 mm) and four times that of RAP (10–20 mm). The price of asphalt (about $450 per ton) is much higher than the price of aggregates (about $10 per ton), so using more FRAP in recycled mixtures can gain more economical benefits.

#### 2.1.2. Aggregates

The aggregates used in this research were limestone produced by the Bei-long-kou Stone Factory in Gansu province (Lanzhou, China). There were five stockpiles with different sizes for aggregates: 0–3 mm, 3–5 mm, 5–10 mm, 10–15 mm, and 15–20 mm. The mineral filler selected was ground limestone. The properties of aggregates and mineral filler were tested according to Chinese specification JTG E42-2005 [19], and the results met the requirements of the Chinese standard JTG F40-2004 [20]. Table 3 and Table 4 show the physical properties of aggregates and mineral filler, respectively.

#### 2.1.3. Virgin Asphalt and Rejuvenator

Ding-bang rejuvenator produced in Shanxi province was selected to recover aged asphalt among RAP due to there being more than 30% RAP in the recycled mixture. Table 5 shows the physical properties of Ding-bang rejuvenator. According to Chinese specification JTG F41-2008 [17], the rejuvenator is blended into the aged asphalt with different doses, and penetration of the recovered binder was tested. The optimal dose was determined by the interpolation method according to the required penetration. Simultaneously, the softening point and ductility of these mixes were also considered. Based on the above operational procedures, the optimal rejuvenator dose was 6% by the weight of aged asphalt in RAP. Virgin asphalt selected for mixture design was SK70#. Its properties were tested according to Chinese specification JTG E20-2011 [21], and the results are shown in Table 6.

### 2.2. Mixture Design

Dense-graded asphalt concrete with a nominal maximum size (NMAS) of 19 mm (AC-20) was selected in this study. There were four percentages of RAP (0, 30%, 40%, and 50% by the weight of total aggregates), and three FRAP contents (10%, 15%, and 20% by the weight of total aggregates) considered for each RAP rate. In order to reduce the influence of the different gradations on the performance of the mixture, this paper selected the median limit gradation of AC-20 specified in the Chinese standard JTG F40-2004 [20] as the target gradation, making the gradation of the mixtures as close as possible to the target level. The material composition ratios of mixtures containing RAP and FRAP are shown in Table 7 and composite gradations of mixtures with various percentages of RAP and FRAP are shown in Table 8.

The Marshall Mix design procedure was applied for mixture design. For recycled mixtures, the preparation of the Marshall specimens were similar to that of virgin mixtures. The main difference was the mixing process. First, RAP and virgin aggregates were mixed at 170–180 °C for 90 s. Second, the virgin asphalt and rejuvenator were mixed with the mixtures for another 90 s. Third, the mineral filler was added to the mixing pot and the blending process lasted for a further 90 s. Finally, the Marshall specimens of recycled mixtures were gained.

The volumetric and mechanical properties of recycled mixtures with five asphalt contents were investigated, including the bulk specific gravity, air voids (VV), voids in mineral aggregate (VMA), voids filled with asphalt (VFA), Marshall stability (MS), and flow value (FV). The target VV was 4.0% and the optimal asphalt content (OAC) could then be calculated according to Chinese standard JTG F40-2004 [20]. The volumetric and mechanical properties of recycled mixtures with different RAP and FRAP contents are shown in Table 9.

### 2.3. Mixture Performance Testing

The modulus of recycled mixtures containing RAP was improved by increasing the RAP content [11], resulting in a better high-temperature performance than that of virgin mixtures. Therefore, this paper investigated the low-temperature, fatigue, and moisture performances of mixtures with different percentages of RAP and FRAP.

#### 2.3.1. Low-Temperature Bending Test

The low-temperature bending test was used to determine the mechanical properties of the flexural failure of HMA at a specified temperature and loading rate. Slabs with a geometric size of 300 mm (length) × 300 mm (width) × 50 mm (height) were produced by a wheel tracking device and then cut into small beam specimens with dimensions of 250 mm (length) × 30 mm (width) × 35 mm (height). There were four specimens for each mixture. The test temperature was −10 °C and loading rate was 50 mm/min. The operating procedure of the test was based on T 0715-2011 in Chinese specification JTG E20-2011 [20].

#### 2.3.2. Semi-Circular Bending Test

There are many testing methods used to evaluate the fatigue performance of asphalt mixtures, including the indirect tensile test, Texas overlay test, semi-circular bending test (SCB), bend beam fatigue test, simplified viscoelastic continuum damage fatigue test, and direct tension test [22]. This paper selected the semi-circular bending (SCB) test to evaluate the potential for fatigue damage of mixtures due to the following advantages.

- Specimen is easy to be produced and just a conventional test system rather than a special device is needed;
- It is also suitable for field performance evaluation;
- A coefficient of variation (COV) of the test was less than 20% [22];
- It had a high repeatability of experimental results [23,24,25].

Previous research [26] has demonstrated that when the thickness of the specimens was greater than 40 mm, the COV decreased sharply. China’s standard [20] stipulates that the thickness of asphalt pavement should be greater than or equal to 2.5–3.0 times as large as the NMAS of aggregates (19 mm × 2.5 = 47.5 mm, the NMAS in this study is 19 mm). Based on the above factors, this study determined that the thickness of the semi-circular specimens was 50 mm.

In order to simulate the compaction on site, ensure the uniformity of the specimens, and reduce the influence of the specimens on the results, cylindrical specimens with the OAC reported above were fabricated by a superpave gyratory compactor and the target VV was 4.0%. The gyration number used to prepare cylindrical specimens was 50 and the VV of specimens was controlled through adjusting the weight of mixtures. The cylindrical specimens were then cut into a smaller size with dimensions of 150 mm (diameter) × 50 mm (height). Subsequently, each smaller specimen was cut in half to form two semi-circular samples, and a notch along the symmetry axis with the size of 15 mm in length and 1.5 mm in width was cut for each semi-circular sample. After that, the semi-circular specimens were tested at 15 °C with a loading rate of 50 mm/min under the conditions of two points bending, and the distance between these two points at the bottom was 120 mm. There were twelve specimens for each mixture. Three specimens among them were used to determine the maximum stress when fracture occurred. Subsequently, the other nine specimens were tested under the condition with a cyclic load at different stress ratios (0.2, 0.3, and 0.4, the ratio of the applied maximum stress to the maximum stress of instantaneous damage), and there were three specimens for each stress ratio. Finally, the fatigue life of mixtures with different stress ratios could be obtained. The correlation between the logarithm of fatigue life and stress ratios showed a linear trend, as described by the following equation [27]:

(1) $$\mathrm{lg}N_{f}=K-n(\frac{\sigma }{s})$$

where *N_f_* is the fatigue life (in cycles); σ/*S* is the stress ratio, indicating the ratio of the applied maximum stress to the maximum stress when fracture occurred; *K* is the intercept of the linear equation, reflecting the fatigue life, and the fatigue life increases with increasing *K*; and *n* is the slope of the linear equation, representing resistance to fatigue damage, and the resistance to fatigue damage decreases with increasing *n*.

#### 2.3.3. Moisture Susceptibility Test

The freeze-thaw splitting test is widely used to evaluate the moisture damage of a mixture. The test was carried out in accordance with T 0729-2000 in Chinese specification JTG E20-2011 [20]. Eight Marshall specimens compacted 50 times for each side were selected and divided into two groups for each mixture. One group was stored at room temperature in a dry environment. The other group was first saturated in a vacuum environment with 97.3–98.7 kPa, and then placed in a freezer at −18 °C for 16 h. After that, the samples were thawed in a 60 °C water bath for 16 h. Finally, both groups were immersed in a water bath at 25 °C for 2 h and then subjected to a splitting test. The tensile strength ratio (TSR) can be calculated by the Equation (2).

(2) $$\mathrm{TSR}\;=\;100\left(\frac{R_{T2}}{R_{T1}}\right)$$

where *R_T_*_2_ is the average tensile strength under freeze-thaw conditions and *R_T_*_1_ is the average tensile strength in a dry environment.

## 3. Results and Discussions

### 3.1. The Effect of RAP and FRAP on Asphalt Content

**Figure 1:**
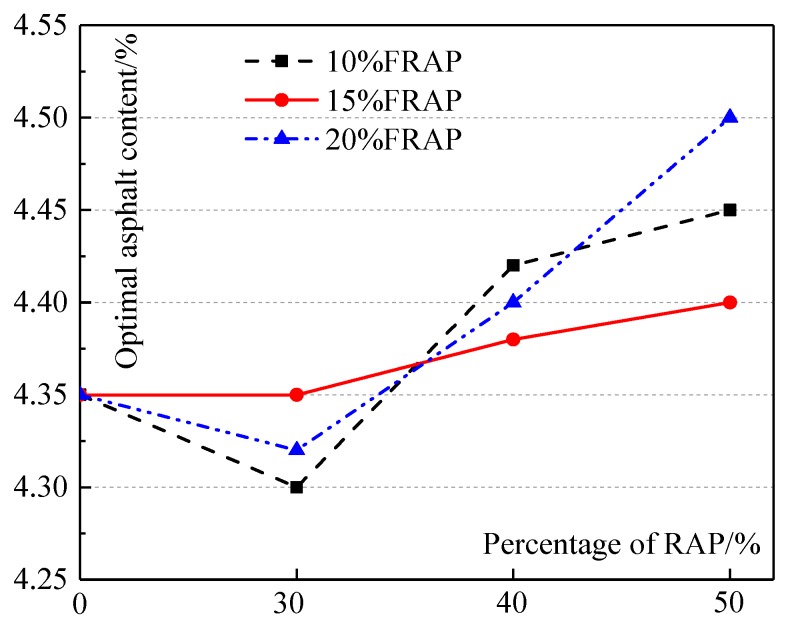
The optimal asphalt content (OAC) of mixtures.

**Figure 2:**
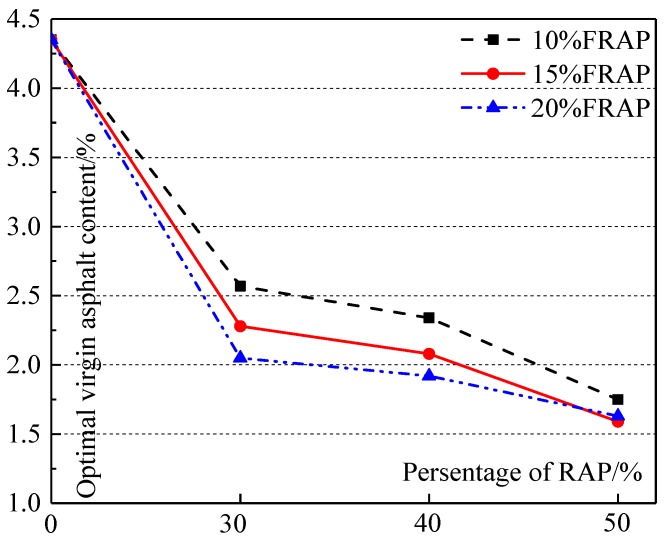
The virgin asphalt content used in recycled mixtures.

The OAC of mixtures and virgin asphalt content used in recycled mixtures are shown in Figure 1 and Figure 2, respectively.

From Figure 1, it can be seen that the OAC of the recycled mixture with 30% RAP is no more than that of the virgin mixture. That is possibly because, compared with the virgin mixture, the recycled mixture (30% RAP) significantly reduces the virgin fine aggregates (0–3 mm) content, being most sensitive to asphalt content. For recycled mixtures, OAC grows slowly in general as the RAP content increases. The previous research divided the aged asphalt in RAP into two kinds. One kind of aged asphalt had no blending with virgin asphalt, acting as black rock; the other kind was total blending with virgin asphalt [3]. With increasing RAP, although the virgin fine aggregate (0–3 mm) contents among recycled mixtures were not significant, the useless aged asphalt (black rock) content grows. Therefore, more virgin asphalt is required to wrap aggregates for mixtures with similar composite gradations, resulting in higher asphalt contents. However, the growth of asphalt contents between virgin mixtures and recycled mixtures is slight, and the maximum value is only about 4.5%. 

Figure 2 shows that the virgin asphalt content used in recycled mixtures decreases with increasing RAP. The usable residual asphalt content in RAP increases as the percentage of RAP grows, so the virgin asphalt content used in a recycled mixture decreases. Compared with the virgin mixture without RAP, 41% (30-R-10) to 63% (50-R-15) of virgin asphalt can be saved with increasing RAP. Up to 29% (30-R-20) of virgin asphalt can be saved with increasing FRAP content for the specified RAP content. When the percent of RAP is up to 50%, the effect of FRAP contents on virgin asphalt contents used in recycled mixtures is the lowest, and the content saved is only 6%.

### 3.2. Low-Temperature Bending Test Analysis

**Figure 3:**
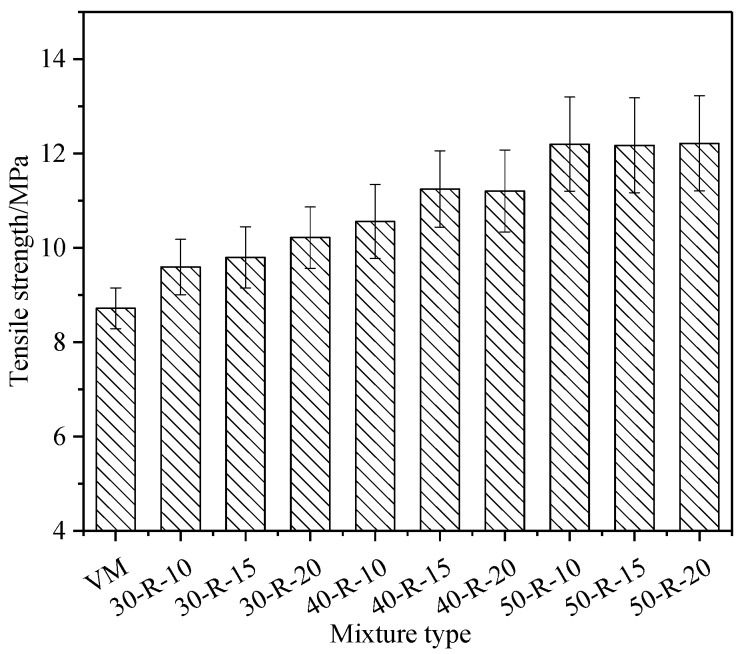
The tensile strength of mixtures with different reclaimed asphalt pavement (RAP) and fine reclaimed asphalt pavement (FRAP) contents.

**Figure 4:**
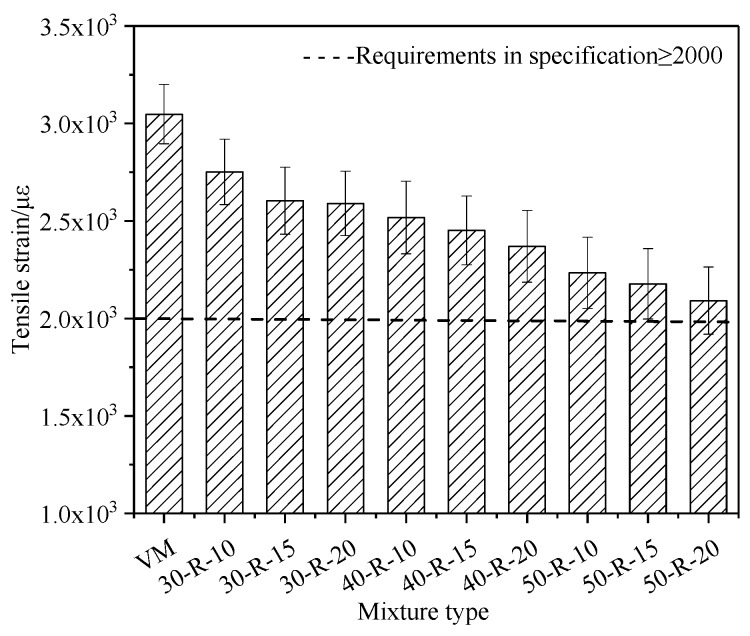
The tensile strain of mixture with different reclaimed asphalt pavement (RAP) and fine reclaimed asphalt pavement (FRAP) contents.

The low-temperature bending test was used to evaluate the properties of mixtures at a low temperature. The test temperature was −10 °C and the loading rate was 50 mm/min. Figure 3 and Figure 4 show the low-temperature bending test results of mixtures with various percentages of RAP and FRAP.

With the increasing RAP and FRAP contents, the tensile strength of mixtures generally increases, while the tensile strain decreases. It indicates that the resistance to load increases, but the resistance to deformation is reduced for mixtures. 

In order to better understand the above results, the modulus of mixtures was investigated by an Indirect Tensile (IDT) test, and the results are shown in Table 10. From Table 10, it can be seen that with increasing RAP and FRAP contents, the modulus of mixtures becomes greater, indicating that mixtures become brittle and hard, hereby resulting in increasing sensitivity of the temperature. The previous findings [28] showed that the technical properties of asphalt binder had a contribution rate of 80% to the low-temperature performance of the mixtures. Although RAP binder can be partially recovered with rejuvenator, the useless residual asphalt content grows relatively with increasing RAP and FRAP. When it accumulates a certain amount, the low-temperature of the mixtures (50-R-20) is close to the limit requirements of the specification.

Statistical analysis of the low-temperature performance of mixtures with different RAP and FRAP contents was performed. This paper defined RAP and FRAP as factors, and defined tensile strength and tensile strain as dependent variables. Two-way ANOVA and Least Significance Difference (LSD) were selected to be performed at a 95% confidence level. The results of ANOVA show that the RAP content in a mixture has a significant effect on the tensile strength and strain, and the FRAP content in the mixture has an insignificant effect on the tensile strength and strain. The results of LSD show consistent conclusions with ANOVA analysis.

### 3.3. Semi-Circular Bending Test Analysis

#### 3.3.1. Fatigue Life at Different Stress Ratios

**Figure 5:**
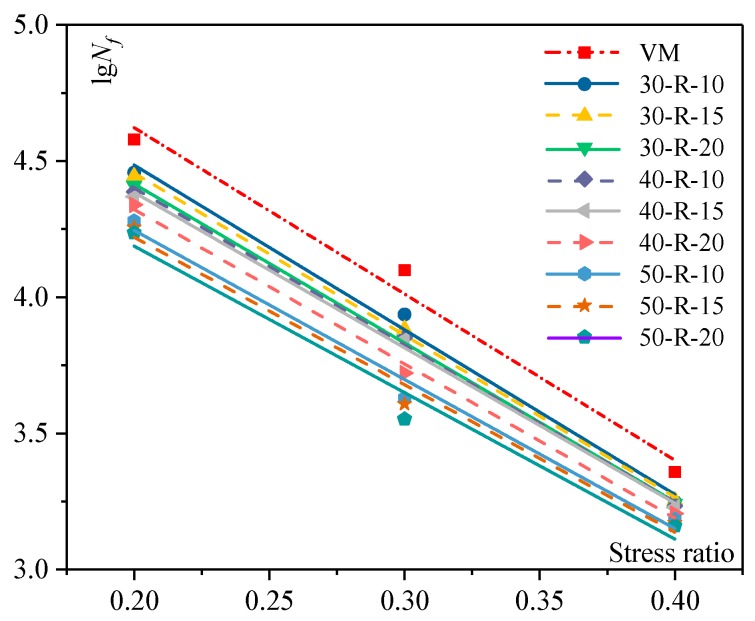
Fatigue life at different stress ratios.

An SCB test was used to evaluate the fatigue resistance of mixtures. The test temperature was 15 °C and the loading rate was 50 mm/min. The fatigue life at different stress ratios with various percentages of RAP and FRAP is shown in Figure 5 and Table 11.

For the same mixture, the fatigue life decreases gradually as the stress ratio increases. This is mainly because the damage of the mixture at large stress ratios during the fatigue test is greater than the damage of the mixtures at a relatively smaller stress ratio. The damage accumulates with repeated loading, which leads to a reduction of the number of cycles required for the mixture with the same damage at a larger stress ratio, so the fatigue life is reduced. 

At the same stress ratio, the fatigue life of the mixture decreases with increasing RAP and FRAP contents. The difference of fatigue life for various mixtures become small with increasing stress ratios. It indicates that fatigue damage of the mixtures more easily occurs at a higher stress ratio.

*K* is the intercept of the fitted equation, which characterizes the fatigue life of the mixture. The fatigue life is greater with a larger *k* value. Compared with the virgin mixture, the *k* value of the mixture gradually decreases with an increasing RAP and FRAP content, resulting in a decreased fatigue life. However, attenuation of the fatigue life is not large in general.

*n* is the slope of the fitted equation, characterizing the sensitivity of the changing rate of fatigue life for mixtures. The changing rate of fatigue life is faster with greater values of *n*. Compared with the virgin mixture, the *n* value of the recycled mixture gradually decreases as the RAP and FRAP content increases, indicating that the recycled mixture with higher RAP and FRAP contents is less sensitive to fatigue damage at different stress ratios. 

The fatigue damage of the mixtures refers to the ability of the mixtures to resist deformation under repeated loads. The modulus of mixtures increases with increasing RAP and FRAP contents. A higher modulus leads to a higher sensitivity for fatigue cracking. Therefore, the fatigue life of the recycled mixtures is lower than that of the virgin mixture, and the change of fatigue life in recycled mixtures with the larger RAP and FRAP content is greater.

#### 3.3.2. Fatigue Life with Different Loads

**Figure 6:**
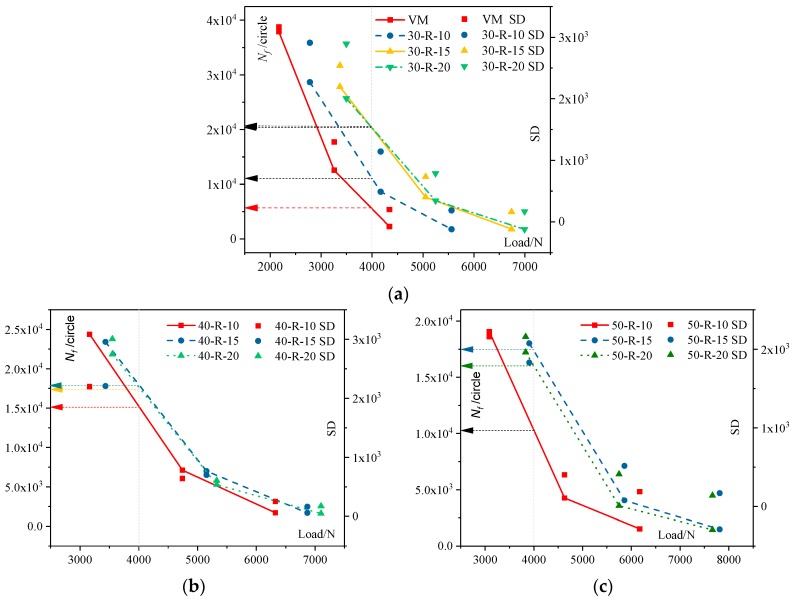
The load and fatigue life between (**a**) the virgin asphalt mixture and recycled mixture with 30% reclaimed asphalt pavement (RAP), (**b**) recycled mixture with 40% RAP, and (**c**) recycled mixture with 50% RAP.

As mentioned before, the modulus of recycled mixtures grows with increasing RAP and FRAP contents, leading to higher failure loads of recycled mixtures than virgin mixtures. The maximum failure loads of recycled mixtures with RAP and FRAP is about 1.3–2.0 times that of virgin mixtures. The recycled mixture is subjected to a larger cyclic load than the virgin mixture during the fatigue testing. It is unreasonable to only analyze the fatigue life at different stress ratios. Therefore, this paper analyzed the fatigue life of different mixtures with repeated loading, and the results are shown in Figure 6. In the figure, *N_f_* is the fatigue life in cycles, and standard deviation is named SD.

The coefficient of variation of all the tests for each specimen varies between 8.4% and 14.8%. SD varies between 140 and 3166 and decreases with increasing stress ratios. It can be seen that with the same cyclic loading, the recycled mixtures seem to have a better fatigue life than the virgin mixes. When the load is 4000 N, the fatigue life of the virgin mixture is 5694 times greater, while the fatigue life of all recycled mixture is more than 10,000 times greater. The fatigue life of the recycled mixture is improved with increasing FRAP contents. However, when the FRAP content is up to 20%, the fatigue life of its mixture is slightly lower than that of the mixture with 15% FRAP for each specific RAP content. From Table 1, it can be drawn that the differences of graduations between FRAP and FRAP aggregates is significant. Therefore, more FRAP contents may result in greater variability for the properties of the mixtures, such as composite gradations or VV of mixtures. The variability in mixtures has a significant effect on the fatigue life. Therefore, this paper recommends that the FRAP content is no more than 15%.

### 3.4. Moisture Susceptibility Test Analysis

**Figure 7:**
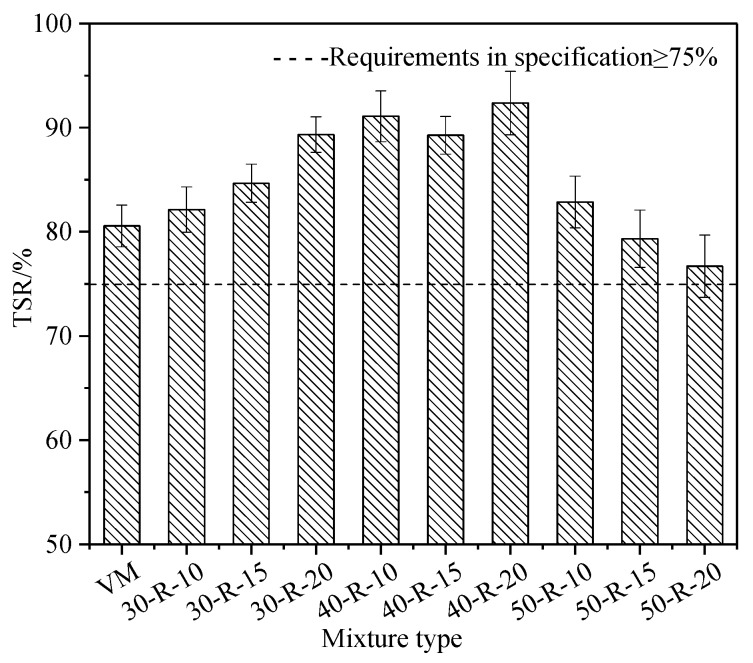
The tensile strength ratio (TSR) of mixtures with various reclaimed asphalt pavement (RAP) and fine reclaimed asphalt pavement (FRAP) contents.

The TSR of mixtures with various RAP and FRAP content are shown in Figure 7.

As the RAP and FRAP contents increase, the TSR of different kinds of mixtures generally exhibits in the form of a parabola. TSR firstly increases and then decreases. RAP aggregates were covered with the aged binder. Virgin aggregates were also more or less covered with RAP binder when they were firstly mixed with RAP during mixing. Therefore, the bond between aggregates and asphalt in recycled mixtures is stronger than that in the virgin mixture. It effectively prevents asphalt stripping off from aggregates. Therefore, recycled mixtures with appropriate amounts of RAP (no more than 40%) have a better moisture damage resistance than virgin mixtures. However, the mixture contains 50% RAP, and its moisture susceptibility is equivalent to or less than that of virgin mixtures. Quantitative characterization between RAP binder and virgin binder was drawn by Zhao et al. [29]. The mobilization rate of RAP binder decreased with increasing RAP contents. When the RAP content reached 50%, the mobilization rate of RAP binder was less than 50%, resulting in a large amount of useless aged binder (black rock). More block rock may lead to higher VV in recycled mixtures (seen in Table 9), making recycled mixtures more sensitive to moisture damage. 

Statistical analysis similar to low-temperature performance was conducted for moisture. The results of ANOVA for moisture performance show the same trend as that of low-temperature performance. The moisture damage resistance significantly changes with diverse RAP contents, while the moisture damage resistance insignificantly changes with different FRAP contents. 

## 4. Conclusions

This paper investigated laboratory performances of mixtures with different percentages of RAP and FRAP, including the low-temperature, fatigue, and moisture performance. The following conclusions can be drawn according to the test results:

(1) The OAC of recycled mixtures increases slightly with increasing percentages of RAP and FRAP. Moreover, the amount of virgin asphalt used in recycled mixtures is significantly reduced as the RAP and FRAP content increases, resulting in up to 63% savings compared with virgin mixtures;
(2) With the increasing RAP and FRAP contents, the tensile strength of mixtures generally increases, while the tensile strain decreases. The low-temperature performance of recycled mixtures (50-R-20) is close to the limit requirements of the specification. The results of ANOVA show that the RAP content in the mixture has a significant effect on the tensile strength and strain, while the effect of FRAP content in mixtures on tensile strength and strain is insignificant;
(3) The correlation between the logarithm of fatigue life and stress ratios shows a linear trend. For the same mixture, the fatigue life decreases gradually as the stress ratio increases. The fatigue life of mixtures decreases with increasing RAP and FRAP contents at the same stress ratio. With increasing RAP and FRAP contents, the fatigue life of mixtures decreases, while the resistance to fatigue damage seems to be improved;
(4) The recycled mixtures seem to have a better fatigue life than the virgin mixes with the same cyclic loading. For the specified RAP content, the fatigue life of the recycled mixture improved with increasing FRAP contents. However, when the FRAP content is up to 20%, the fatigue life of its mixture is slightly lower than that of the mixture with 15% FRAP, indicating that the FRAP content should be no more than 15%;
(5) As the RAP and FRAP contents increase, the TSR of different kinds of mixtures is generally exhibited in the form of a parabola. The TSR firstly increases and then decreases. The recycled mixture (40-R-20) has the best moisture damage resistance. The results of ANOVA show that the effect of different RAP contents on the moisture damage resistant is significant, while the moisture damage resistance insignificantly changes with different FRAP contents.

The recycled mixtures not only save the amount of virgin asphalt, but also have excellent laboratory performances meeting the requirements of the specifications, although the field performances of recycled mixtures with high RAP and FRAP still need to be further confirmed.

## Figures and Tables

**Table 1:** The gradations of reclaimed asphalt pavement (RAP).

Sieve Size/mm		26.5	19.0	16.0	13.2	9.5	4.75	2.36	1.18	0.6	0.3	0.15	0.075
10–20 mm	a	100	92.8	80.5	55.6	27.8	17.3	13.3	11.4	8.9	6.8	4.2	2.6
	b	100	64.5	32.8	7.7	0.6	0.2	0.2	0.2	0.2	0.2	0.2	0.2
5–10 mm	a	100	100	97	92.6	85.3	62.7	39.6	31.2	23.5	18.4	12.9	8.6
	b	100	100	100	95	83.3	43.4	17	10.1	3.2	0.5	0.2	0.1
FRAP	a	100	100	100	100	100	99.7	95.1	84.6	59.9	39.7	24.9	17.5
	b	100	100	100	100	100	98.6	87	69.2	33.9	5.4	0.9	0.2

Note: a refers to RAP containing the aged binder and RAP aggregates; b only refers to RAP aggregates. FRAP: Fine reclaimed asphalt pavement.

**Table 2:** Asphalt content and physical properties of aged asphalt binder among reclaimed asphalt pavement (RAP).

Asphalt Content of Each Type RAP			Physical Properties of Aged Asphalt Binder		
Fine Reclaimed Asphalt Pavement FRAP %	5–10 mm %	10–20 mm %	Penetration at 25 °C/0.1 mm	Softening Point (TR&B)/°C	Ductility at 10 °C/cm
8.2	5.1	2.0	32	68	9

**Table 3:** Physical Properties of Aggregates.

Test Items	Value					Technical Requirements
	15–20 mm	10–15 mm	5–10 mm	3–5 mm	0–3 mm	
Apparent relative density	2.894	2.901	2.894	2.872	2.828	≥2.5
Mud content/%	0.3	0.5	0.6	0.9	1.2	≤1 or ≤3
Water absorption/%	0.34	0.52	0.56	—	—	≤3.0
Crushing value/%	15.3	15.3	—	—	—	≤28
Los Angeles abrasion/%	18.5	18.5	—	—	—	≤30
Flat or elongated/%	6.8	8.9	7.6			
Sand equivalent/%	—	—	—	—	72.3	≥60
Angularity/s	—	—	—	—	51.3	≥30

Note: The technical requirement of the mud content for coarse aggregates is no more than 1%, and the value for fine aggregates is no more than 3%.

**Table 4:** Physical Properties of Mineral Filler.

Test Items	Value	Technical Requirements
Apparent relative density	2.691	≥2.5
Water absorption/%	0.2	≤1
Grain sizes <0.6 mm/% <0.15 mm/% <0.075 mm/%	100.0 93.0 88.1	100
		90–100
		75–100
Hydrophilic coefficient	0.60	≤1

**Table 5:** Physical properties of Ding-bang rejuvenator.

Viscosity at 60 °C/cSt	Flash Point /°C	Saturated Content/%	Aromatic Content/%	Viscosity Ratio	Change in Mass Percentage/%
2800	265	26.7	48.2	1.4	−1.3

Note: Viscosity ratio refers to the ratio of viscosity at 60 °C after a Rotated Thin Film Oven Test (RTFOT) to that before RTFOT, and change in mass percentage refers to the ratio of rejuvenator mass percentage after RTFOT to that before RTFOT.

**Table 6:** Physical properties of virgin asphalt binder.

Penetration at 25 °C/0.1 mm	Softening Point (TR&B)/°C	Ductility at 10 °C/cm
64	47.1	>100

**Table 7:** Material composition ratios of mixtures containing various percentages of reclaimed asphalt pavement (RAP) and fine reclaimed asphalt pavement (FRAP).

Mixture Type	RAP			Aggregate					Mineral Filler (%)
	FRAP (%)	5–10 mm/%	10–20 mm/%	15–20 mm/%	10–15 mm/%	5–10 mm/%	3–5 mm/%	0–3 mm/%	
**VM**	0	0	0	25	20	14	8	30	3
30-R-10	10	10	10	20	15	12	8	13	2
30-R-15	15	10	5	21	18	13	7	9	2
30-R-20	20	5	5	20	17	19	7	5	2
40-R-10	10	14	16	15	14	9	10	10	2
40-R-15	15	10	15	17	15	12	9	5	2
40-R-20	20	5	15	16	18	12	7	6	1
50-R-10	10	25	15	15	14	8	5	7	1
50-R-15	15	18	17	13	14	12	5	5	1
50-R-20	20	10	20	16	12	14	5	2	1

Note: VM is virgin mixture without RAP; the front number (30-R-10) indicates the percentage of RAP and the latter number is the percent of FRAP, and so on.

**Table 8:** Composite gradations of mixtures with various percentages of reclaimed asphalt pavement (RAP) and fine reclaimed asphalt pavement (FRAP).

Sieve Size/mm	26.5	19	16	13.2	9.5	4.75	2.36	1.18	0.6	0.3	0.15	0.075
Target gradation	100	95.0	85.0	71.0	61.0	41.0	30.0	22.5	16.0	11.0	8.5	5.0
VM	100	94.8	81.9	71.7	57.2	42.1	25.9	22.2	18.2	15.5	12.8	6.0
30-R-10	100	95.1	83.3	72.3	58.0	41.9	27.0	23.3	18.1	14.1	10.6	5.7
30-R-15	100	95.3	83.5	73.0	57.9	41.4	28.1	24.5	18.7	14.1	10.3	5.8
30-R-20	100	95.5	84.4	74.5	60.5	40.4	28.0	24.7	18.7	13.7	9.7	5.7
40-R-10	100	95.7	85.6	74.5	58.9	43.7	27.1	23.3	18.0	14.0	10.3	5.9
40-R-15	100	95.4	84.5	73.1	57.3	40.7	26.5	23.1	17.6	13.2	9.5	5.7
40-R-20	100	95.6	85.3	73.8	56.4	40.9	29.0	25.4	19.0	13.7	9.4	6.1
50-R-10	100	95.8	85.5	74.1	58.1	42.0	28.0	23.7	18.0	13.7	9.8	5.6
50-R-15	100	96.1	86.7	75.7	59.6	41.6	28.8	24.7	18.6	13.8	9.6	5.5
50-R-20	100	95.2	84.2	72.4	57.4	39.5	28.6	25.0	18.6	13.3	9.0	5.2

**Table 9:** The volumetric and mechanical properties of recycled mixtures with different reclaimed asphalt pavement (RAP) and fine reclaimed asphalt pavement (FRAP) contents.

Mixture Type	OAC	VV/%	VMA/%	VFA/%	MS/kN	FV/0.1 mm
VM	4.35%	4.48	14.48	69.03	11.35	30.5
30-R-10	4.30%	4.09	13.69	70.15	12.03	29.1
30-R-15	4.35%	4.10	13.90	70.55	12.38	28.2
30-R-20	4.32%	4.26	14.22	70.07	12.23	30.7
40-R-10	4.42%	4.19	14.12	70.36	12.17	30.2
40-R-15	4.38%	4.42	14.53	69.55	12.26	29.4
40-R-20	4.40%	4.39	14.71	70.14	12.55	27.3
50-R-10	4.45%	4.41	14.94	70.48	12.30	27.6
50-R-15	4.40%	4.51	14.63	69.19	12.45	30.1
50-R-20	4.50%	4.53	14.86	69.50	12.74	28.6

**Table 10:** The modulus of mixtures with different reclaimed asphalt pavement (RAP) and fine reclaimed asphalt pavement (FRAP) contents.

Mixtures Type	VM	30-R-10	30-R-15	30-R-20	40-R-10	40-R-15	40-R-20	50-R-10	50-R-15	50-R-20
Modulus /MPa	0.43	0.46	0.53	0.63	0.57	0.65	0.75	0.75	0.84	0.97

**Table 11:** Regression parameter between the logarithm of fatigue life and different stress ratios.

Parameters	VM	30-R-10	30-R-15	30-R-20	40-R-10	40-R-15	40-R-20	50-R-10	50-R-15	50-R-20
k	5.844	5.695	5.646	5.583	5.554	5.526	5.456	5.341	5.304	5.263
n	6.106	6.047	5.95	5.837	5.766	5.706	5.67	5.476	5.419	5.3785
